# Temporally-Controlled Site-Specific Recombination in Zebrafish

**DOI:** 10.1371/journal.pone.0004640

**Published:** 2009-02-27

**Authors:** Stefan Hans, Jan Kaslin, Dorian Freudenreich, Michael Brand

**Affiliations:** Biotechnology Center and Center for Regenerative Therapies Dresden, Dresden University of Technology, Dresden, Germany; Institute of Infectious Disease and Molecular Medicine, South Africa

## Abstract

Conventional use of the site-specific recombinase Cre is a powerful technology in mouse, but almost absent in other vertebrate model organisms. In zebrafish, Cre-mediated recombination efficiency was previously very low. Here we show that using transposon-mediated transgenesis, Cre is in fact highly efficient in this organism. Furthermore, temporal control of recombination can be achieved by using the ligand-inducible CreER^T2^. Site-specific recombination only occurs upon administration of the drug tamoxifen (TAM) or its active metabolite, 4-hydroxy-tamoxifen (4-OHT). Cre-mediated recombination is detectable already 4 or 2 hours after administration of TAM or 4-OHT, demonstrating fast recombination kinetics. In addition, low doses of TAM allow mosaic labeling of single cells. Combined, our results show that conditional Cre/lox will be a valuable tool for both, embryonic and adult zebrafish studies. Furthermore, single copy insertion transgenesis of Cre/lox constructs suggest a strategy suitable also for other organisms.

## Introduction

Zebrafish *(Danio rerio)* is a widely used model organism for vertebrate development and disease [1] due to its short generation time, large number of offspring and optical clarity which allow large-scale forward mutagenesis screens with relative ease [2], [3]. Techniques for reverse genetic analysis are available in zebrafish including morpholino-mediated gene knock-down [4], TILLING [5] and targeted mutagenesis using zinc-finger nucleases [6], [7]. A complementary reverse genetic application is the temporal and spatial controlled over-expression of genes using the Gal4-UAS [8] and the mifepristone-inducible LexPR system [9]. However, control of the gene of interest is conferred by the presence of a transcriptional activator, Gal4FF and LexPR, respectively. Once the transcriptional activator has vanished, expression of the gene of interest is lost, impeding genetic fate mapping in zebrafish. In mouse the most common method for genetic fate mapping is the use of site-specific recombinases such as Cre and Flp [10], [11]. Cre promotes strand exchanges between two 34-bp loxP target sites without any additional cofactors. Each loxP site consists of two 13-bp repeats flanking an 8-bp asymmetric spacer sequence that confers directionality. Head-to-head orientation causes inversion of the DNA between the two sites, whereas head-to-tail orientation results in the excision of the intervening DNA sequence, an irreversible reaction due to the loss of the excised product. Based on the observation that the localization of proteins can be controlled by a ligand when fused to a ligand-binding domain of a steroid hormone receptor, chimeric Cre recombinases were developed [12]. This approach offers temporal control of Cre-mediated recombination and allows targeting late aspects of a dynamic or broad Cre expression. Currently, Cre fused to the mutated human ligand-binding domain of the estrogen receptor (CreER^T2^) has the best properties for ligand sensitivity and inducible recombination efficiency [13].

Previous attempts to establish the Cre/loxP system in zebrafish showed its general functionality in this organism [14], [15] but recombination was very inefficient [16], [17]. In one of these studies, the recombination efficiency was evaluated in embryos at 24 hours post fertilization (hpf); only 129 recombined cells per animal could be detected although the recombined transgene was driven by the broad *β-actin* promoter and high, ubiquitous levels of Cre were provided during gastrulation [16]. Currently, the reason for the recombination inefficiency is unknown, but the method by which transgenic zebrafish were generated, might be crucial. Until recently, the method of choice remained the injection of plasmid DNA into the cytoplasm of one cell-stage embryos resulting in concatemeric DNA integration of up to 2000 copies [18], [19]. Copy number can be significantly lowered by meganuclease-mediated transgenesis [20], but unambiguous site-specific recombination requires single copy insertions which can only be achieved by pseudotyped retrovirus [21] or transposon-mediated transgenesis [22].

## Results

**Figure 1:**
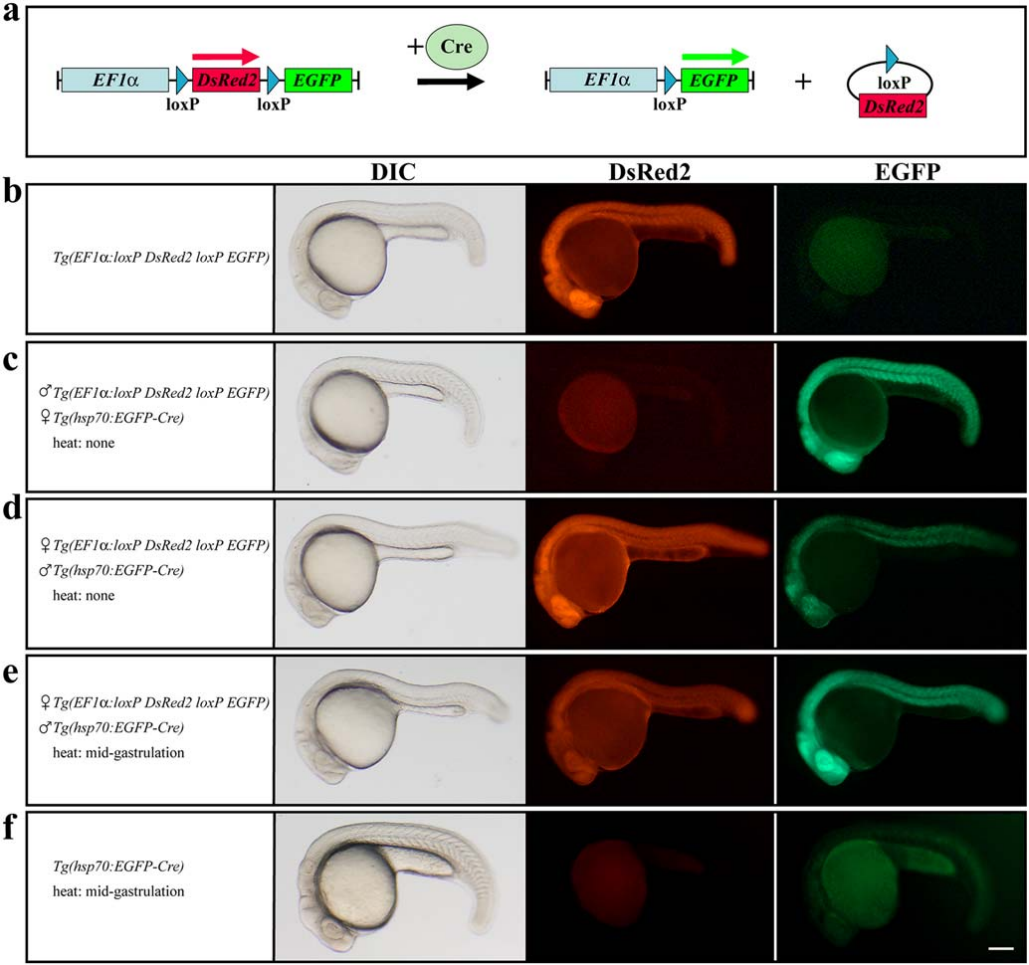
Cre-mediated recombination in the red-to-green reporter line. (a) Scheme of the recombination event. In the absence of Cre the *EF1*α promoter drives the expression of DsRed2 but changes to EGFP after successful Cre-mediated recombination. (b) Embryos of the red-to-green reporter line show strong DsRed2 and no EGFP fluorescence. (c) Maternal contribution of the *Tg(hsp70:EGFP-Cre)* allele results in complete loss of DsRed2 and ubiquitous EGFP expression in double transgenic embryos. (d) Paternal contribution of the *Tg(hsp70:EGFP-Cre)* allele leads to strong DsRed2 and mosaic EGFP expression in double transgenic embryos. (e) Paternal contribution of the *Tg(hsp70:EGFP-Cre)* allele and brief heat induction at mid-gastrulation stages results in reduced DsRed2 and strong ubiquitous EGFP expression in double transgenic embryos. (f) Embryos of the *Tg(hsp70:EGFP-Cre)* line show only weak EGFP fluorescence after brief heat induction at mid-gastrulation stages. b–f Lateral views of live 24 hpf embryos bearing different transgenes. Scale bar, 125 µm.

Here, we used the Tol2 transposon system to generate a red-to-green reporter line for easy detection of Cre activity. In the absence of Cre activity the reporter line expresses DsRed2 under the control of the *Xenopus Elongation Factor 1 alpha* (*EF1*α) promoter, but changes to EGFP after a successful recombination event (Fig. 1a,b). When crossed to the *Tg(hsp70:EGFP-Cre)* [14] line which carries conventional Cre fused to EGFP under the control of the zebrafish temperature-inducible *hsp70* promoter, we observe Cre-mediated recombination in a sex-specific manner even at permissive temperatures. If the *Tg(hsp70:EGFP-Cre)* allele is maternally provided, double transgenic embryos at 24 hpf show complete loss of DsRed2 and ubiquitous EGFP expression, indicating recombination in all cells (Fig. 1c). In contrast, paternal contribution of the *Tg(hsp70:EGFP-Cre)* allele retains the strong DsRed2 signal but nevertheless leads to significant EGFP expression in a mosaic fashion (Fig. 1d). However, following a brief heat induction at mid-gastrulation stages that results in only weak EGFP fluorescence derived from the *Tg(hsp70:EGFP-Cre)* allele, the DsRed2 signal is reduced and strong ubiquitous EGFP expression can be observed in double transgenic embryos (Fig. 1e,f). Analysis by *in situ* hybridization shows absence of DsRed2 transcripts in these embryos (data not shown). This indicates that the observed fluorescent DsRed2 is maternally provided as well as translated from transcripts made prior the recombination event. Although native EGFP fluorescence could not be observed, our results suggest a basal leakiness of the *hsp70* promoter in the *Tg(hsp70:EGFP-Cre)* line at permissive temperatures which was already reported for the similar *Tg(hsp70:Cre)* line [16], [17]. When we crossed our red-to-green reporter line with *Tg(hsp70:Cre)* the results corroborated our previous finding with one exception. Less maternal contribution is observed and double transgenic embryos always display a strong DsRed2 signal and significant EGFP expression in a mosaic fashion at 24 hpf. Again, brief heat induction at mid-gastrulation stages leads to reduced DsRed2 and strong ubiquitous EGFP expression at 24 hpf (data not shown) indicating successful recombination events in most or all cells. Our data show that Cre is highly efficient in zebrafish as the basal leakiness of the *hsp70* promoter is already sufficient to induce significant recombination. This is in contrast to previous findings resulting in low recombination frequencies even in the presence of high Cre expression levels [16], [17]. In both studies, effector lines were generated by plasmid injection, whereas we used transposon-mediated transgenesis. In agreement with that, we also observed no or only poor recombination with other stable red-to-green reporter lines that we generated by plasmid injection (data not shown). Consequently, the transgenesis approach might be crucial as only single copy insertions allow unambiguous Cre-mediated excision events.

**Figure 2:**
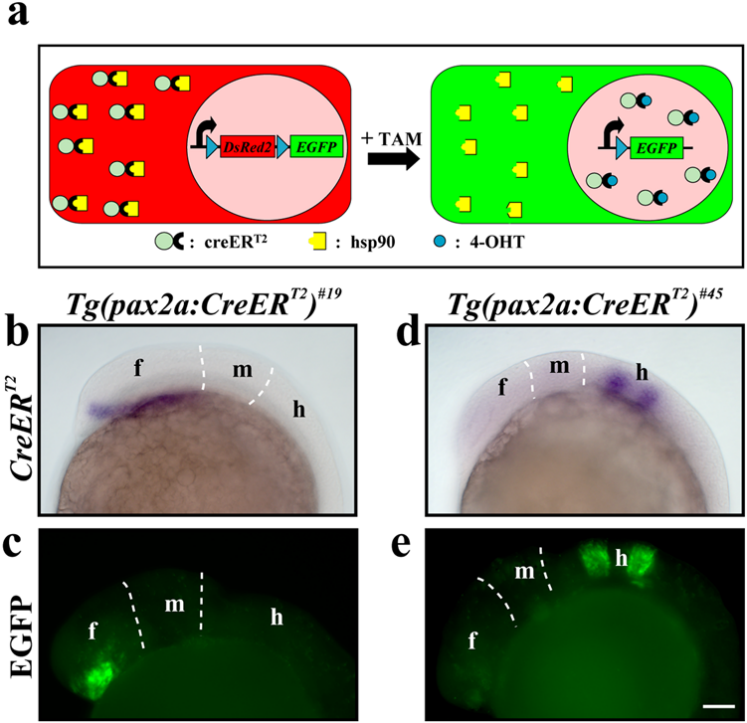
Ligand-dependent Cre-mediated recombination. (a) Scheme of the ligand-dependent recombination event in cells of the red-to-green reporter line. The chimeric CreER^T2^ recombinase is retained in the cytoplasm in the absence of the ligand. After administration of TAM which is converted to the active ligand 4-OHT, CreER^T2^ translocates to the nucleus, where it catalyzes the recombination event. (b) Expression of CreER^T2^ in the diencephalon of the *Tg(pax2a:CreER^T2^)^#19^* line at early segmentation stages revealed by *in situ* hybridization. (c) EGFP expression in the diencephalon of double transgenic embryos at 24 hpf bearing the red-to-green reporter and the *Tg(pax2a:CreER^T2^)^#19^* alleles after TAM treatment at mid-gastrulation stages. (d) Expression of CreER^T2^ in rhombomere 3 and 5 of the *Tg(pax2a:CreER^T2^)^#45^* line at early segmentation stages revealed by *in situ* hybridization. (e) EGFP expression in rhombomere 3 and 5 of double transgenic embryos at 24 hpf bearing the red-to-green reporter and the *Tg(pax2a:CreER^T2^)^#45^* alleles after TAM treatment at mid-gastrulation stages. Abbreviations: f, forebrain; h, hindbrain; m, midbrain. Scale bar, 50 µm.

Because the leakiness of the *hsp70* promoter results in non-conditional recombination, we turned towards using the tamoxifen-inducible CreER^T2^ recombinase (Fig. 2a). We generated transgenic lines driving CreER^T2^ under the control of the zebrafish *pax2a* promoter [23] and established two lines that express CreER^T2^ transcripts only in a subset of the reported pattern presumably due to position effects. In *Tg(pax2a:CreER^T2^)^#19^* CreER^T2^ is expressed in the prospective diencephalon of the developing forebrain whereas in *Tg(pax2a:CreER^T2^)^#45^* it is expressed in the future rhombomere 3 and 5 of the hindbrain anlage (Fig. 2b,d). When crossed to our red-to-green reporter line, a strong EGFP signal is detected in the diencephalon or in rhombomere 3 and 5 in double transgenic embryos at 24 hpf (Fig. 2c,e). Recombination was very robust, with EGFP recapitulating the entire *CreER^T2^*-positive domain of all double transgenic embryos. However, different intensities in the EGFP signal indicate that Cre recombinase-mediated recombination does not happen at the same time in all the cells of the *CreER^T2^*-positive domain. The detection was strictly dependent on CreER^T2^ activation by tamoxifen (TAM), which was applied at mid-gastrulation stages. We never observe any EGFP expression in the absence of TAM indicating that CreER^T2^ is successfully retained in the cytoplasm. Thus, only administration of the ligand results in the translocation of the recombinase to the nucleus, where it catalyzes the recombination event. However, high levels of CreER^T2^, supplied by mRNA injection or the *hsp70* promoter in a stable transgenic line, can lead to non-conditional recombination (data not shown), suggesting that very high levels of CreER^T2^ can overwhelm the cellular machinery.

**Figure 3:**
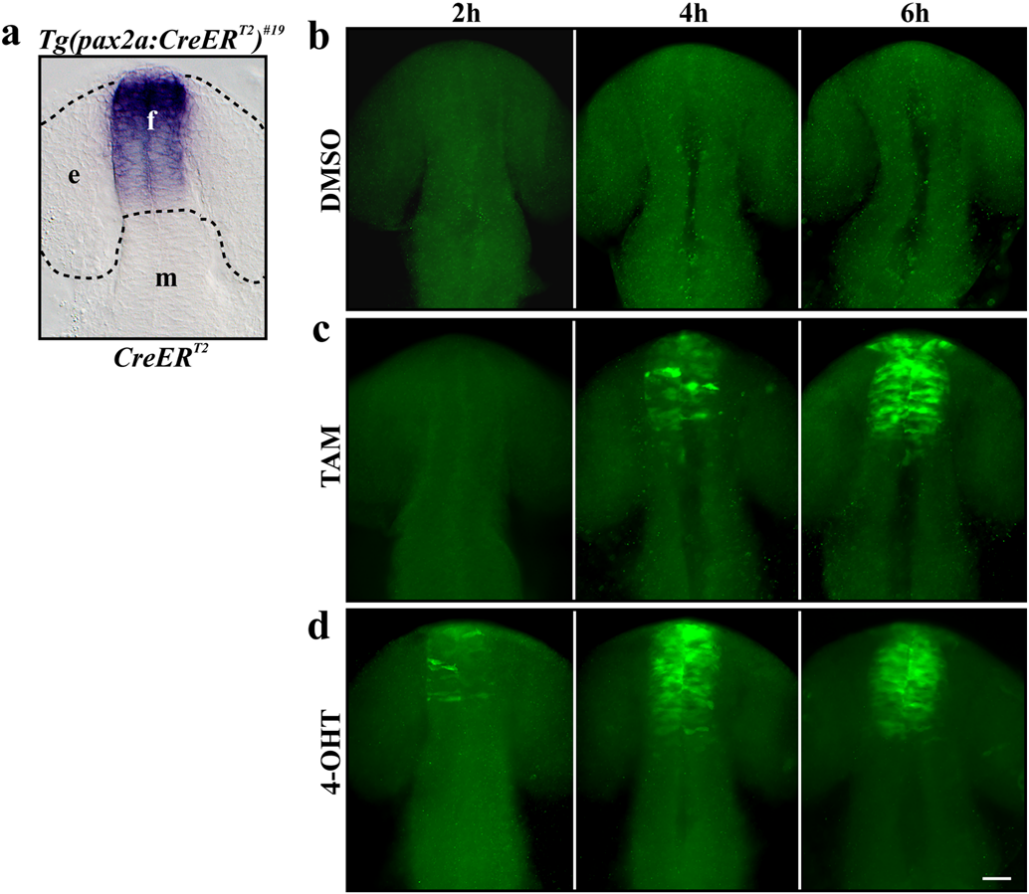
Kinetics of ligand-dependent Cre-mediated recombination. (a) Expression of CreER^T2^ in the *Tg(pax2a:CreER^T2^)^#19^* line at the 12-somite stage revealed by *in situ* hybridization. (b) Control embryos treated with DMSO never show any EGFP. (c) Immunofluorescence staining with antibodies to EGFP is detectable 4 hours after application of TAM and expanded further after 6 hours. (d) Onset of EGFP expression by immunofluorescence staining is detected after 2 hours and expanded further after 4 and 6 hours after application of 4-OHT. a–d Dorsal views of double transgenic embryos at 12-, 16-, 20 and 24-somite stage (15, 17, 19 and 21 hpf). Abbreviations: f, forebrain; e, eye anlage; h, hours; m, midbrain. Scale bar, 30 µm.

We next addressed the kinetics of CreER^T2^-mediated recombination in zebrafish embryos. In mouse, TAM is most commonly used to induce recombination. However, the active metabolite that binds to the mutated ligand-binding domain of the human estrogen receptor is 4-hydroxy-tamoxifen (4-OHT). Hepatic conversion of TAM to 4-OHT results in a 6–12 hour time delay between administration of TAM and onset of recombination [24]. To determine the time delay in zebrafish embryos, we crossed our red-to-green reporter line with homozygous *Tg(pax2a:CreER^T2^)^#19^* carriers which render all DsRed2-positive embryos also positive for the CreER^T2^ driver. At the 12-somite stage when CreER^T2^ is expressed strongly in the diencephalon of the developing forebrain (Fig. 3a) we applied DMSO, TAM or 4-OHT and fixed embryos after 2, 4 and 6 hour intervals, respectively. The EGFP signal was detected by immunofluorescence staining with an anti-GFP antibody, as EGFP-fluorescence requires a further 90 min to 4 h after protein synthesis until the mature chromophore is folded [25]. As mentioned above, embryos never showed any EGFP signal without TAM treatment (Fig. 3b). However after administration of TAM, positive recombination was already detectable after 4 hours whereas widespread EGFP expression could be observed after 6 hours (Fig. 3c). Onset of recombination was even faster in embryos treated with 4-OHT. EGFP-positive cells were already visible after 2 hours and widespread EGFP expression was detected after 4 and 6 hours (Fig. 3d). In comparison to the EGFP signal detected by immunofluorescence staining, native EGFP fluorescence was observed 6 and 4 hours after the administration of TAM and 4-OHT, respectively (data not shown). In addition to the faster kinetics of 4-OHT, we also observe that 4-OHT is more potent since the effective concentration of 4-OHT with 0.5 µM is one magnitude lower than the concentration of TAM (5 µM). Taken together, our results show that ligand-mediated recombination is not only feasible but also surprisingly fast in the developing zebrafish embryo. Furthermore, as the ligand is simply administered to the medium the recombination event can be precisely timed depending on the experimental goal.

**Figure 4:**
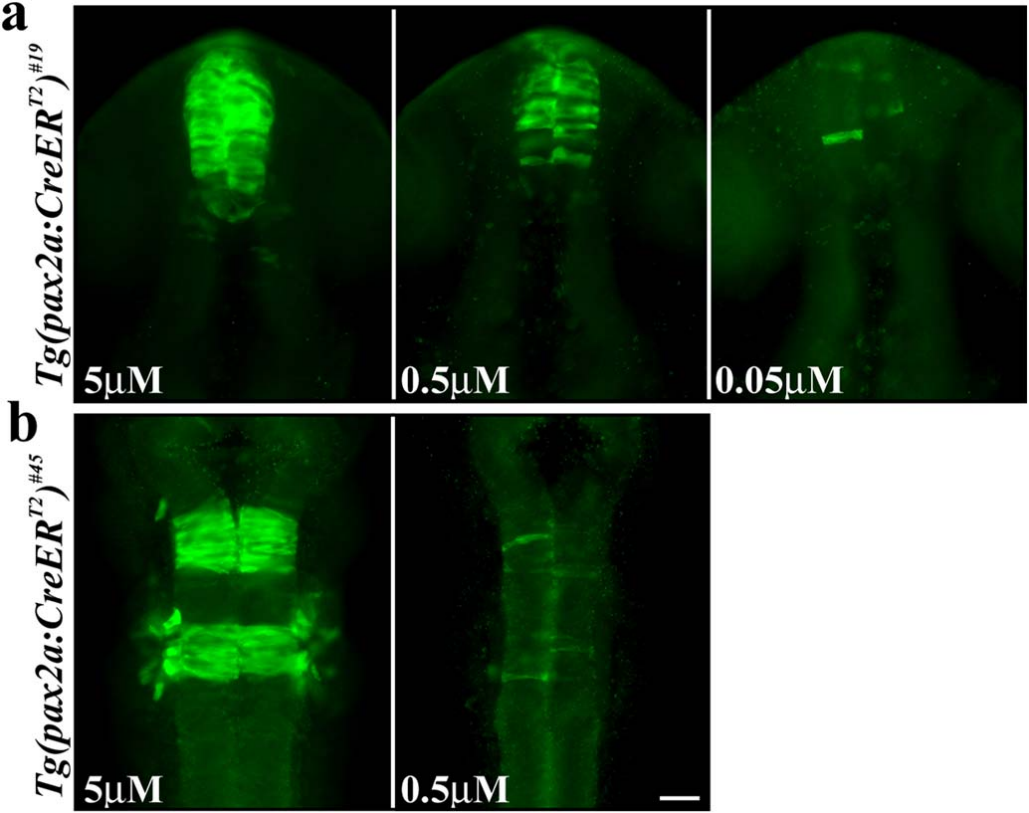
Dose-dependent recombination in the *Tg(pax2a:CreER^T2^)^#19^* and *Tg(pax2a:CreER^T2^)^#45^* lines by TAM. (a) Double transgenic embryos bearing the red-to-green reporter and the *Tg(pax2a:CreER^T2^)^#19^* alleles show strong EGFP expression in the diencephalon after application of 5 µM TAM at mid-gastrulation stages. Application of 0.5 µM TAM or 0.05 µM TAM at the same stage, results in reduced EGFP expression or single EGFP-positive cells, respectively. (b) Double transgenic embryos bearing the red-to-green reporter and the *Tg(pax2a:CreER^T2^)^#45^* alleles show strong EGFP expression in rhombomere 3 and 5 after application of 5 µM TAM at mid-gastrulation stages. Application of 0.5 µM TAM at the same stage, results in single EGFP-positive cells. a, b Dorsal views of double transgenic embryos at 24 hpf. Scale bar, 30 µm.

Although most experiments may aim at maximal recombination, graded levels or even single recombined cells might be preferred for certain studies. In mouse it has been shown that the rate of recombination can be controlled by the dose of the ligand administered [24]. Because the ligand controls the translocation of the recombinase to the nucleus, lower ligand concentrations result in less available nuclear CreER^T2^ protein. Consequently, the probability of recombination between the target loxP sites decreases. In order to test this hypothesis in zebrafish we crossed our red-to-green reporter with the *Tg(pax2a:CreER^T2^)^#19^* and *Tg(pax2a:CreER^T2^)^#45^* lines and exposed the progeny to different TAM concentrations. TAM was applied at mid-gastrulation stages and EGFP was detected by anti-GFP antibody staining in embryos fixed at 24 hpf. Our toxicity analysis revealed aberrant development and embryonic lethality with TAM concentrations of 10 µM and higher (data not shown). Thus our experiments were carried out with 5 µM TAM representing maximal recombination in proper developing embryos. However, after lowering TAM concentrations we observe different outcomes for the *Tg(pax2a:CreER^T2^)^#19^* and *Tg(pax2a:CreER^T2^)^#45^* lines, respectively. Double transgenic embryos carrying the reporter and the *Tg(pax2a:CreER^T2^)^#19^* allele show lower but still substantial EGFP expression at 0.5 µM TAM and single recombined cells can be observed at 0.05 µM TAM (Fig. 4a). In contrast, combination of the *Tg(pax2a:CreER^T2^)^#45^* allele with the reporter line results in single recombined cells at a concentration of 0.5 µM TAM (Fig. 4b). As our *in situ* analysis revealed higher CreER^T2^ expression levels in the *Tg(pax2a:CreER^T2^)^#19^* line than in the *Tg(pax2a:CreER^T2^)^#45^* line, we assume that the difference in CreER^T2^ expression levels is responsible for the observed rate of recombination at lower TAM concentrations. Combined, our data show that graded levels and even single recombination events can be achieved. However, TAM conditions depend on the expression strength of CreER^T2^ and hence need to be tested and optimized for each CreER^T2^ driver line.

## Discussion

In principle, the use of our red-to-green reporter line should allow genetic fate mapping in zebrafish in the same manner as in mouse. In mouse ubiquitous expression is most commonly achieved by the use of the Rosa26 locus [26], whereas we use the *EF1*α promoter which has been reported to show high and ubiquitous expression in zebrafish [22]. However, although we see strong ubiquitous expression on the protein level for up to 7 days, our *in situ* analysis reveals strong ubiquitous expression only during gastrulation and mid-somitogenesis stages (data not shown). Subsequently, expression is shut down in a tissue-specific manner with strong transcription maintained only in the retina and mid-hindbrain boundary beyond 24 hpf (data not shown). Expression analysis of the *EF1*α promoter in the adult brain also revealed promoter activity in a non-ubiquitous fashion (data not shown). Thus, the *EF1*α promoter can be a valuable tool for embryonic studies but further promoters need to be tested for actual ubiquitous expression in zebrafish matching the usefulness of the mouse Rosa26 locus. However, reporter genes driven by existing, tissue-specific promoters should already allow the labeling of smaller or even single gene expression domains in zebrafish.

Our work demonstrates the efficient use of the conditional Cre/lox system in zebrafish. It will allow temporal and spatial overexpression studies and facilitate *bona fide* lineage tracing of cell fates. In addition, it will provide the opportunity to develop recombinase-mediated techniques for genomic manipulations. These include the generation of conditional knockout alleles which are currently unavailable in zebrafish. Targeted mutagenesis using zinc-finger nuclease involves a double-strand break that is repaired by nonhomologous end-joining [6], [7]. The direct ligation of the two DNA strands frequently results in the loss or gain of small amounts of DNA sequence. If an exogenous repair template is also supplied, the induced double-strand break can be repaired by homology-directed repair and sequence alterations encoded in the repair template are incorporated into the genome [27]. In this way loxP target sites could be introduced to flank a gene of interest which would allow the temporally controlled inactivation of the gene by Cre-mediated recombination.

Finally, our results suggest that the use of site-specific recombinases may be applicable to many other organisms that can be subjected to single-copy transposon-mediated transgenesis [28] as a prerequisite for efficient, unambiguous Cre-mediated excision.

## Materials and Methods

### Plasmid construction

To create the pTol *EF1*α loxP-DsRed2-loxP EGFP plasmid, three elements (the *EF1*α promoter, the loxP-DsRed2-loxP cassette and the EGFP gene) were PCR-amplified flanked by unique restriction sites that allow easy substitution of one of the three elements in the final vector. The following enzymes were used: *XhoI* and *FseI* for the *EF1*α promoter, *FseI* and *SmaI* for the loxP-DsRed2-loxP cassette and *SmaI* and *AscI* for the EGFP gene. The products were sequentially subcloned into the *XhoI*/*AscI* site of the pTol2000 [22] vector which results in the opposite orientation of the Tol2 transcription. The pTol *pax2a:CreER^T2^* plasmid was generated in the same manner. The following enzymes were used: *SalI* and *FseI* for the *pax2a* promoter [23] and *FseI* and *AscI* for the CreER^T2^ gene [12]. Again the products were subcloned into the *XhoI*/*AscI* site of the pTol2000^22^ vector as *SalI* and *XhoI* produce compatible cohesive ends.

### Zebrafish husbandry and germ line transformation

Zebrafish embryos were obtained by natural spawnings of adult AB wild-type fish maintained at 28.5°C on a 14 hour light, 10 hour dark cycle and staged as described [29]. For germ line transformation, plasmid DNA and transposase mRNA were injected into fertilized eggs (F0), raised to adulthood and crossed to AB wild-type fish as previously described [22]. For the red-to-green reporter line, F1 embryos were examined under a fluorescent microscope and positive embryos were raised. This way, ten independent F0 were identified and tested for recombination in the presence of Cre. As they all showed efficient recombination, the strongest allele was chosen to establish the red-to-green reporter line which now shows efficient recombination in the third generation. For the *pax2a:CreER^T2^* line, F1 embryos were screened by PCR using *pax2a* (ttgccaacgttgtaggctactacc)- and Cre (tagagcctgttttgcacgttcacc)-specific primers that result in an 867 base pair fragment. Positive F0 were re-crossed, fixed at 24 hpf and expression of CreER^T2^ was analyzed by *in situ* hybridization. Out of 8 PCR-positive F0 fish, only two lines, *Tg(pax2a:CreER^T2^)^#19^* and *Tg(pax2a:CreER^T2^)^#45^* showed a distinctive CreER^T2^ expression pattern. Both lines were established and carriers are identified by either PCR or cross to the red-to-green reporter line.

### Immunocytochemistry and *in situ* hybridization

Antibody staining was carried out as described [30]. Antibodies were used in the following concentrations: α-GFP (Molecular Probes), 1∶500; goat α-rabbit Alexa Fluor 488 (Molecular Probes), 1∶500. Embryos were analyzed using a Zeiss Axiophot 2 microscope. For probe synthesis the CreER^T2^ gene was subcloned into the CS2+ vector, linearized with BamHI and transcribed with T7. Probe synthesis and *in situ* hybridization was performed essentially as previously described [30].

### Pharmacological treatments and brief heat induction

For pharmacological treatments the following stock solutions were made and stored at −20°C: 50 mM tamoxifen (TAM; Sigma, T5648) in DMSO; 25 mM 4-hydroxy-tamoxifen (4-OHT; Sigma, H7904) in ethanol. For embryo treatments, dilutions of these chemicals were made in embryo medium as follows: TAM: 5, 0.5 and 0.05 µM; 4-OHT: 0.5 µM. At mid-gastrulation (75% epiboly or 8 hpf) or at 12-somite stage (15 hpf) embryos, still in their chorions, were transferred into petri dishes containing the treatment solution. For control treatments, sibling embryos were incubated in corresponding dilutions of DMSO and ethanol. All incubations were conducted in the dark. For brief heat induction, undechorionated embryos at mid-gastrulation stages were transferred into fresh petri dishes. After removal of excess embryo medium, 42°C embryo medium was added and the petri dishes were returned to the 28.5°C incubator. This heat induction protocol results in only weak EGFP fluorescence at 24 hpf when applied to the *Tg(hsp70:EGFP-Cre)* [16] line at mid-gastrulation stages.
